# Network Analysis of MERS Coronavirus within Households, Communities, and Hospitals to Identify Most Centralized and Super-Spreading in the Arabian Peninsula, 2012 to 2016

**DOI:** 10.1155/2018/6725284

**Published:** 2018-05-07

**Authors:** Oyelola A. Adegboye, Faiz Elfaki

**Affiliations:** ^1^Australian Institute of Tropical Health and Medicine, James Cook University, Townsville, QLD 4811, Australia; ^2^Department of Mathematics, Statistics and Physics, Qatar University, Doha 2713, Qatar

## Abstract

Contact history is crucial during an infectious disease outbreak and vital when seeking to understand and predict the spread of infectious diseases in human populations. The transmission connectivity networks of people infected with highly contagious Middle East respiratory syndrome coronavirus (MERS-CoV) in Saudi Arabia were assessed to identify super-spreading events among the infected patients between 2012 and 2016. Of the 1379 MERS cases recorded during the study period, 321 (23.3%) cases were linked to hospital infection, out of which 203 (14.7%) cases occurred among healthcare workers. There were 1113 isolated cases while the number of recorded contacts per MERS patient is between 1 (*n*=210) and 17 (*n*=1), with a mean of 0.27 (SD = 0.76). Five super-important nodes were identified based on their high number of connected contacts worthy of prioritization (at least degree of 5). The number of secondary cases in each SSE varies (range, 5–17). The eigenvector centrality was significantly (*p* < 0.05) associated with place of exposure, with hospitals having on average significantly higher eigenvector centrality than other places of exposure. Results suggested that being a healthcare worker has a higher eigenvector centrality score on average than being nonhealthcare workers. Pathogenic droplets are easily transmitted within a confined area of hospitals; therefore, control measures should be put in place to curtail the number of hospital visitors and movements of nonessential staff within the healthcare facility with MERS cases.

## 1. Introduction

Middle East respiratory syndrome coronavirus (MERS-CoV) is a contagious respiratory pathogen that is contracted via close contact with infected individuals. Interactions among individuals can aid super-spreading of infectious diseases in humans or animals, and it is usually highest among individuals in close proximity with one another. MERS-CoV was first reported in a 60-year-old man in Bisha area of the Kingdom of Saudi Arabia in 2012 [1]. It has now spread across 27 countries in 4 continents. The most index case-patients have either resided in or have travelled to areas neighbouring the Arabian Peninsula [2]. From the intermittent transmission that had occurred in animal-to-human [1, 3], many human-to-human cases have also been documented within families and healthcare facilities [3–7].

During an infectious disease outbreak such as MERS-CoV (or MERS for short), the transmission usually forms networks of infected individuals (cluster of outbreaks) probably because of the way the virus crosses from one infected individual to another susceptible individual. In other words, the source of infection may be the direct or indirect connection [8]. In network analysis, the aim is to identify the most crucial infected patients (also called nodes), who are important in the super-spreading and to use the location of the node in the network to predict which patients (nodes) are likely to be infected [9]. These important patients are influential in that they infect disproportionately large numbers of secondary contacts [10, 11].

Super-spreading events (SSEs) are as a result of individuals (agents of SSE) harbouring the virus who infect disproportionately more secondary contacts, as compared to most others [7, 10, 11]. Therefore, an SSE consists of a large cluster of infection in which some individuals infect many more other individuals, thereby acting as agents for an SSE. In this study, super-spreading was defined as having at least five contacts. SSEs have been documented in other infectious disease outbreaks such as SARS in Beijing 2003 [12] and more recently Ebola in West Africa, 2014–2015 [13, 14], and MER-CoV in South Korea, 2015 [10].

The knowledge of people's connectivity network is very crucial in the spread of infectious diseases transmitted via pathogenic droplets such as respiratory infection, MERS, and Ebola. As in the case of MERS, occasional larger cluster sizes should not be unexpected, such as the outbreak in South Korea [7]. Although there have been variations in the size of human-human transmission of MERS, its high variability and heterogeneity in the transmission potential have been underscored [15, 16].

Over the years, researchers have been exploring how the knowledge of network structures could influence public health measures. For example, network analysis based on connectivity centrality was used to identify high-risk people for targeted vaccination in an effort to contain the spread of infectious disease [17]. Network analysis was used to investigate whether the density of the network contacts of persons infected by *Mycobacterium tuberculosis* was more likely to be tested positive for tuberculosis (TB) compared to the occurrence of TB clusters detected through network connections with clusters detected by molecular genotyping [18]. Similarly, the effect of protein-protein interactions within the host-pathogen interactome was explored via network analysis on pathogen fitness during infection [19].

The focus of this study was to map and measure relationships and flows between people infected with MERS and to investigate the structure of MERS transmission with the help of network and graphs. Patient's interactions and links were obtained through contact tracing within 14 days prior to the onset of the disease. The structure of network connectivity will assist in identifying the most influential contacts, while network centrality metrics were used to investigate the contribution and characteristics of the agents of super-spreading to the infection and spread of MERS. And lastly, the role played by patient's attributes (node property) was investigated as an epidemic amplifier or attenuator via hypotheses testing.

## 2. Materials and Methods

### 2.1. Data Sources

This study is based on case-by-case list of clinical-confirmed MERS cases provided by Dr. Rambaut [20]. The focus is on the 1379 MERS cases reported in the Kingdom of Saudi Arabia between June 2012 and September 2016. The variables considered in this study were age, gender, patient type (whether the patient is a healthcare worker (HCW) or nonhealthcare worker), health outcome (dead or alive) as at the last day of follow-up, patient comorbidity status, types of exposure to known risk factors (animal contact and camel contact indirectly or directly or through consumption of camel products), and place of infection (classified as hospital, community, and household/family). The data set was cross validated with information from WHO disease outbreak news and Saudi Arabia MOH MERS command and control website.

### 2.2. Study Area

**Figure 1:**
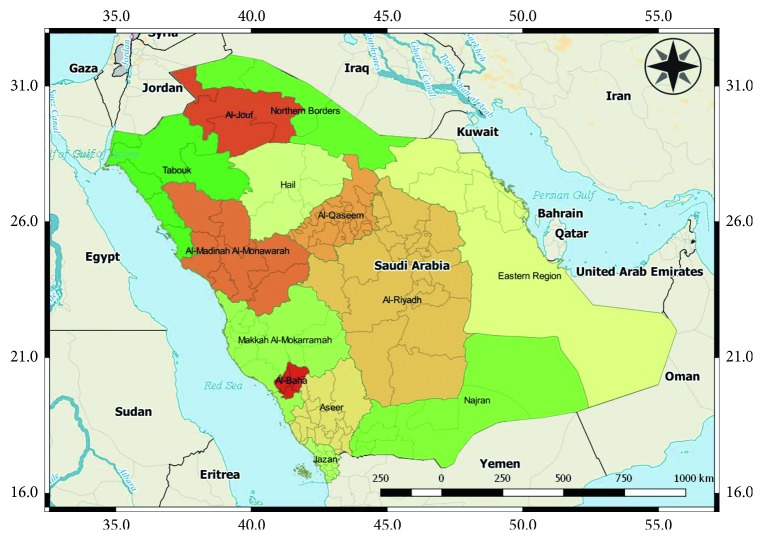
Map showing the Kingdom of Saudi Arabia and surrounding countries.

The Kingdom of Saudi Arabia (KSA) is the main part of the peninsula bounded by the Red Sea on the west, Gulf of Aden on the south, Gulf of Oman on the south east, and Arabian Gulf on the east. The KSA measures about 2,150,000 square kilometres and shares its border with several countries, such as Jordan and Iraq in the north, Qatar, Bahrain, Kuwait and United Arab Emirates in the east, and Oman and Yemen in the south. The country is situated on latitude 15.66–32.15 and longitude 34.5–55.67 (Figure 1). The KSA is divided into 13 regions (Manatiq, administrative level 1) and 118 governorates (Muhafazat, administrative level 2). The World Bank as of 2014 estimated its population to be 30.89 million [21].

### 2.3. Definitions, Contact Tracing, and Sociodemographic Variables

MERS patients were clinically confirmed via real-time RNA-positive strand virus using reverse transcription polymerase chain reaction (RT-PCR) with a positive PCR on at least two specific genomic targets, upstream E protein (upE) and ORF1a, or a single positive target (upE) with sequencing of a second target RdRpSeq assay or *N* gene (NSeq assay) [6, 22]. Patients' contact investigation was conducted by hospital officials by tracing patient's history of exposure to other known risk factors such as contact with other laboratory-confirmed MERS cases, animal/camel contact, or visiting other places known to be linked to MERS cases 14 days prior to the onset of symptoms. An animal contact patient implies a patient with historical contact with animals, while camel contact patients were those who work in a camel market or have history of contact with camels, or consumed camel products in 14 days prior to the onset of symptoms.

A healthcare worker is anyone who works in a healthcare facility (all personnel, such as doctors, nurses, laboratory staff, securities, and receptionists). A patient is said to have comorbidity if he or she has coexisting chronic diseases or medical conditions or has been admitted to the hospital due to unrelated medical conditions. Place of exposure is classified as (a) hospital infection if the infection occurred in a hospital, for example, a healthcare worker/hospital visitor/outpatient contracting the disease in a healthcare facilities, (b) family infection if the infection was through a family member or within the household, or (c) community infection if the infection is contracted outside the hospital or household, such as in schools, workplace, hajj tents, etc.

### 2.4. Statistical Analysis

Descriptive analysis was conducted on some sociodemographic variables presented as mean and standard deviation for age and frequencies and percentages for other categorical variables. Prevalence of MERS disease across communities, household, and hospitals was tested via the chi-square statistic for categorical variables while the *t*-test was used for continuous variables (Table 1).

**Figure 2:**
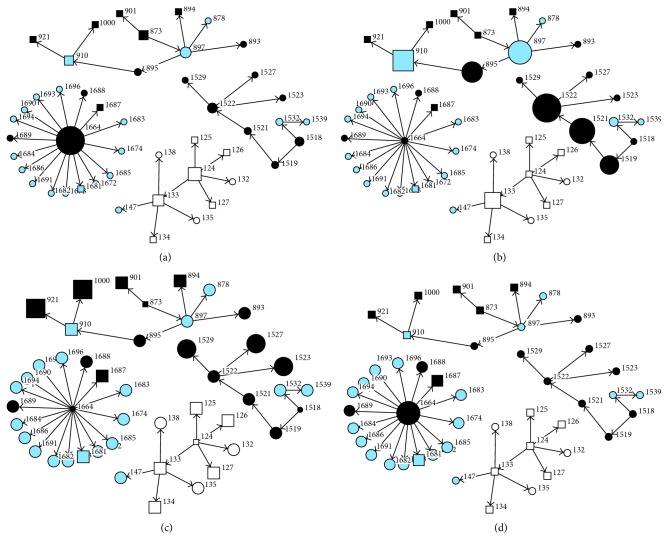
Illustration of (a) degree-, (b) betweenness-, (c) closeness-, and (d) eigenvector-centrality metrics for sample MERS infection network. Each of the patient number represents a node connected by links called edges. The square nodes represent males while the circle nodes represent females. Grey represents healthcare workers and dark colours are nonhealthcare workers while white is unknown. The node sizes indicate the centrality values in Table 2.

The units of network analysis in this study are the nodes representing individuals infected with MERS within families, hospitals, or communities which are connected via edges. The outbreak network visualization and network analyses were conducted in R package “igraph” [23] and UCINET 6.0 Version 1.00 [24]. We used centrality metrics to measure the structural importance of patients (nodes) in a network. The node “degree centrality” was used to reveal the most active nodes in the network and how well a node is connected with its neighbours—a node degree is the number of edge incidents on a node. The “betweenness centrality” was used to measure how many pairs of nodes a node can be connected to through a shortest path, while the “closeness centrality” was used to measure how contagious an infected patient (a node) is to others [9, 17, 24, 25]. Similarly, “2-reach centrality” was used to explore the proportion of nodes that can reach a given node in 2 steps or less while “eigenvector centrality” was used to measure the importance of a node depending on the importance of its neighbours. Figures 2(a)–2(d) provide an illustration of different network centrality metrics.

Finally, the effects of patient's attributes on their position in a network (measured by network centrality metrics) were investigated, and the relationship between two (or more) network centrality metrics was explored. We used permutation tests in UCINET 6.0 Version 1.00 [24] to test specifically the following aspects: (1) whether more central patients are HCWs or not, (2) whether more central infections happened in the hospital or community, and (3) how much of the variation in patient's betweenness centrality, for example, can be explained by their out-degree, for example.

## 3. Results

### 3.1. Descriptive Summaries

**Figure 3:**
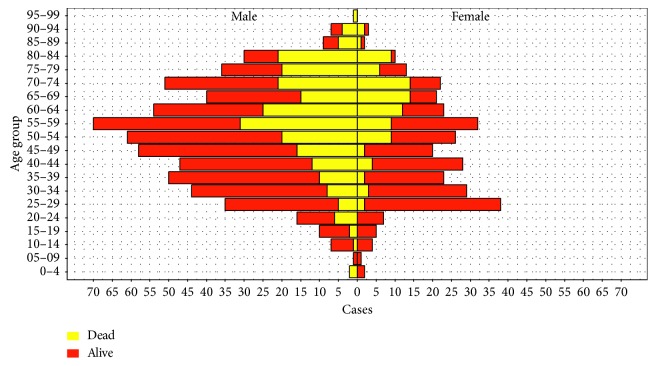
Distribution of MERS-CoV infection in males and females among different age structures in the KSA.

During the study period, 1379 MERS cases were recorded in the KSA, most cases occurred in Ar Riyad (46.7%) and Makkah (24.3%). The overall crude fatality rate (CFR) is 33.79% (466 fatalities in 1379 cases) (Table 1). There were more cases among males (901, 65.3%) than females (460, 33.4%). The mean age of infected MERS patients was 51.7 years, 82% of all cases occurred in people between the ages 25 and 74 years. High proportions of MERS cases were observed among male patients aged 30 years to 74 years (76% of the male cases) and female patients aged 25 years to 74 years (85% of the female cases) (Table 1 and Figure 3).

MERS fatality increases with age, 11% of the infection occurred in older males aged 55–59 years and more fatal in older men (above 50 years). Similarly, 85% of fatality in females occurred in women above 50 years. A high proportion (about 79%) of patients had some kind of prior medical condition (comorbidity). The number of patients with comorbidity varied slightly across the regions. There was higher fatal outcome in patients with comorbidity than patients without any comorbidity. About 47% of those with some kind of medical condition died of the disease compared to only 17% of patients without medical condition.

The results showed that about half of cases (629 (45.6%)) were linked to at least one place of exposure where 321 (23.3%) of them were linked to hospital outbreaks (Table 1). There were 750 (54.4%) cases, whose contact history of infection was unknown. Of the 321 cases linked to hospital outbreaks, more than one-third (*n*=119) occurred among healthcare workers, indicating that about 58.7% of all cases involving healthcare workers happened in the hospital (data not shown in Table 1).

### 3.2. Networks Visualization and Centrality Metrics for the MERS Data

**Figure 4:**
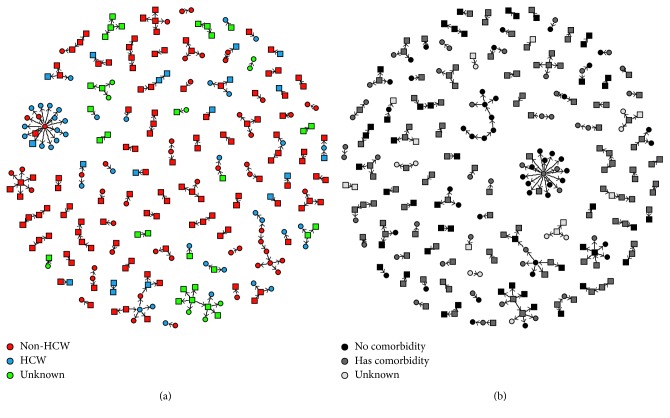
Network contact structure for the spread of MERS-CoV infection in the KSA between 2012 and 2016 showing (a) whether an individual is a healthcare worker or not and (b) individual's comorbidity status. Males are represented by squares while females are represented by circles. Nodes represent all tagged individuals. Isolated cases (nodes of degree zero) are not shown.

The contact structure of MERS cases in the KSA between June 2012 and September 2016 is displayed in Figure 4. Isolated nodes (*n*=1113 (80.7%)) with degree of zero are not shown in the figure. There were 1113 isolated cases while the number of recorded contacts per MERS patient is between 1 (*n*=210) and 17 (*n*=1), with a mean of 0.27 (SD = 0.76). There were a total of 266 connections—110 primary cases and 156 secondary cases. The majority of the first line secondary cases did not produce any further secondary cases of their own. The largest cluster has a wheel-and-spoke configuration in which patient number 1664 is in the centre and linked to 17 other secondary cases (6.4% of the total cases in the network) (Figure 4). The results showed that most of the outbreaks occurred in the hospital (indicated by dark line in Figure 4) and that most of the infected patients were nonhealthcare workers with comorbidity (indicated by green nodes in Figure 4).

Table 2 presents the measures of network centrality estimates for the 10 most important nodes (subsequently called patients) selected by each network metric. A total of 31 nodes worthy of prioritization were selected based on these network metrics (Table 2). It was revealed that 9 out of 31 (29%) of the cases are healthcare workers and 8 (25.8%) cases had some kind of comorbidity. About half of the prioritized nodes (*n*=15 (48.4%)) occurred in hospital settings while 7 (22.6%) cases were fatal.

Based on degree centrality metrics, the top five most important cases were identified based on at least five secondary cases, indicating their high number of connected contacts worthy of prioritization (Table 2). The number of secondary cases in each SSE varies slightly (range, 5–17). Closeness and 2-Reach network metrics ranked the same patients (1664, 1672, 1673, 1674, 1681, 1682, 1683, 1684, 1685, and 1686) among the top 10 cases worthy of prioritization while the eigenvector metrics list was also very similar. The betweenness scores, which indicate the number of other nodes infected by the given node, indicated that patient 1522, 1521, 897, 895, and 910 had the top 5 highest betweenness score of a range of 6 to 9.

**Figure 5:**
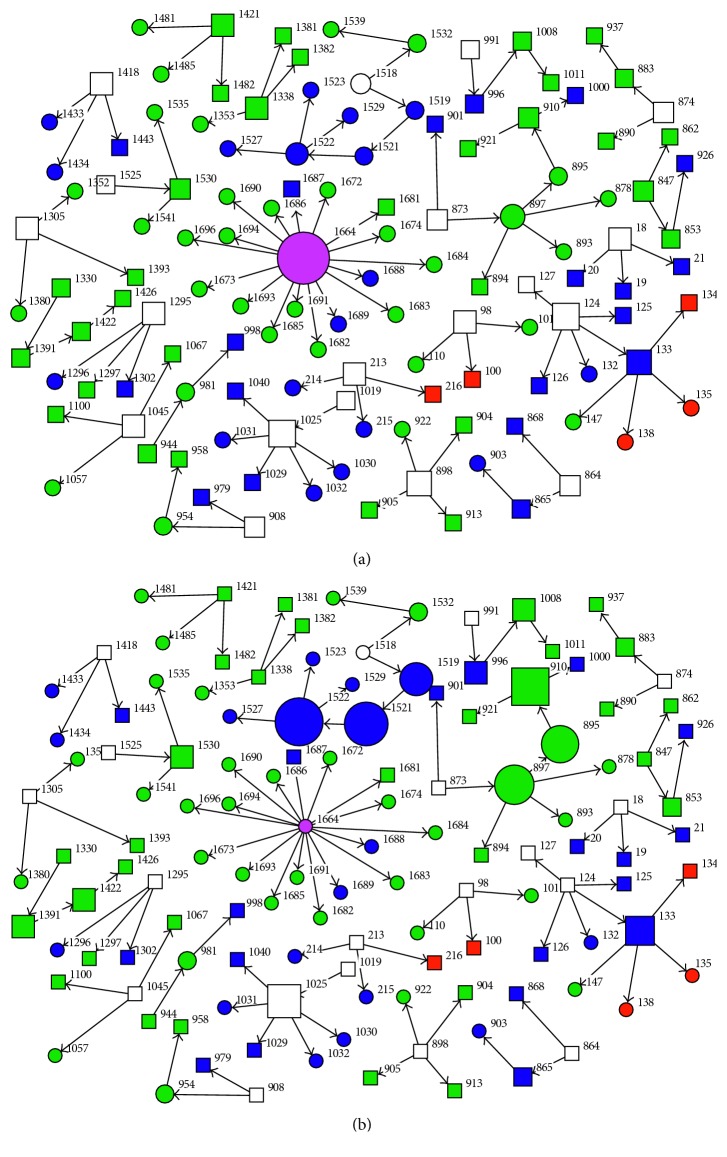
Network contact structure for the spread of MERS-CoV infection in the KSA between 2012 and 2016 for top 10 influential nodes based on degree and betweenness centrality in Table 2. Larger sized nodes implies (a) degree centrality and (b) betweenness centrality. The circle nodes are females while square nodes are males. The node colours represent contact history: pink is the index patient, green is hospital linked, blue is community or family linked, red denotes the primary contact, and white nodes represent unknown contact history.

Among the important cases according to degree centrality, four resulted in fatality. Patient 1664 was favoured (based on degree, closeness, betweenness, and eigenvector network centrality metrics) as the most important in the transmission network by having the highest number of secondary cases. However, the cases selected using betweenness centrality were slightly different from other metrics with patient 1522 topping the list. The degree and betweenness centrality estimates are depicted in Figures 5(a) and 5(b), respectively. The larger node area shows the level of worthiness of prioritization.

### 3.3. Patients Attributes and Network Centrality

When network centrality metrics were used to investigate the association between patient's attributes and patient's position in a network, only eigenvector centrality was significantly associated with two patient's attributes. The eigenvector centrality was significantly (*p* < 0.05) associated with place of exposure, with hospital infection having on average significantly higher eigenvector centrality than other places of exposure. Similarly, being a healthcare worker was significantly associated (*p* < 0.05) with eigenvector centrality. To answer the questions about the relationships among the network centrality metrics, the results suggest that patient's degree centrality explains 90.2% of the variation in patient's closeness centrality, while 14% and 9.3% of the variation in closeness centrality were explained by eigenvector and betweenness centrality, respectively.

Furthermore, we investigated whether super-spreading patients with high network centrality metrics (e.g., degree, betweenness, closeness, eigenvector, and 2-reach) have similar attributes. Significant differences were observed in age, gender, healthcare worker, and fatal cases across the regions; however, no difference was observed in the proportion of patients with comorbidities.

## 4. Discussion

In this study, several network centrality metrics (degree, betweenness, closeness, eigenvector, and 2-reach) were used to quantify the connectivity among MERS cases and to identify which patient requires prioritization for intervention. Usually the patient with the highest degree has the most ties to other patients in the network [9]. Saudi Arabia is said to be facing continuous risk of MERS outbreaks [26]. The findings emphasize the importance of patient's level characteristics in understanding their level of infectiousness. Results show that healthcare facilities and healthcare workers are the most crucial factors in driving national epidemics of MERS. Although healthcare workers are at higher risk of MERS infection due to their proximity to infected patients, previous studies have shown that cases among healthcare workers are less serious [27] with few fatalities [28, 29].

Hospital infections display higher interconnectivity and, on average, are linked to patients that are more connected to highly connected patients than nonhospital infections. Recent studies have reported the outbreaks of MERS in hospitals [5, 30–33]. The overcrowding in hospitals due to easy access to medical care caused MERS to move quickly throughout Korea [5]. Virtually, all cases of MERS in Korea occurred in a hospital-to-hospital type of transmission [5].

Similarly, healthcare workers are more connected to important nodes themselves than nonhealthcare workers. High connectivity among healthcare workers and their proximity to MERS cases is not surprising because of the nature of their job. In general, healthcare workers across Saudi Arabia have negative attitude toward MERS infection [34, 35]. Although infectious disease epidemiological plans were put in place in some hospitals, outbreaks of MERS still occurred due to the failure to adhere to the infection control measures [33]. A high proportion of healthcare workers felt at risk of contracting the disease but obliged to care for MERS patients [33, 34]. Similarly, a high percentage of healthcare workers do not feel safe at work using standard precautions [34].

In terms of the number of connected contacts (via degree centrality), nodes 1664, 1025, 124, 133, and 897 were the top 5 most active MERS cases, but they do not reflect the spread of MERS. The most influential node is patient 1664 based on degree centrality. Most of the secondary cases connected to patient 1664 were either attended to by the same healthcare worker(s) or are themselves healthcare workers that attended to patient 1664. Patients who are strongly tied in a network are more likely to be similar to each other than different. Patient 1664 was a 47-year-old female admitted to the hospital with unrelated symptoms [36], and she was associated with 14 healthcare worker cases and 3 household cases because many healthcare staff treated her at the initial stage during her hospitalization in a vascular surgery ward and initially in an open ward, stressing the importance of limiting access to other patients and quarantines.

Regarding infectivity, when determining the strength of a patient in a network through not only its connectivity but also the interconnectivity of its secondary cases, the betweenness metric provides better estimate. The low betweenness score of patient 1664 indicates that while patient 1664 connects many other patients, it is not a pathway to further infection. Nodes 1522, 1521, 897, 895, and 910 were identified as critical in the spread of MERS to other cases using the betweenness metric. These patients acted as a bridge connecting other smaller secondary cases and thus the spread of MERS infection by further connecting with other secondary cases who also connect other important cases. It was found that the individuals who connect 2 or more separate contacts have an increased likelihood to connect to multiple contacts [37]. Patient 1522 is a 26-year-old female with no comorbidities was linked to three secondary cases and five other indirect offspring.

The major limitation in this study lies in the data sources and contact tracing accuracy. The analysis is based on retrospective data rather than prospective data collected from multiple sources which are publicly available. The accuracy of some of the information provided by the patient may not be verifiable especially during the early outbreaks; however, the reporting has been improved upon over the years with coordination between Saudi Ministry of Health and regional WHO office. Similarly, while it is acknowledged that most reported clusters occurred in the hospital as a result of contact tracing, this is not surprising because of adequate monitoring and data collection in the hospital that revealed large secondary cases in the healthcare facilities. Lastly, as in the case of publicly available data, these study data are characterized by missing data. While the problems associated with inference-drawn, publicly available epidemiological data are acknowledged, the results are meaningful and suggest that structure and characteristics of contact network can indeed have significant effect on the rate of transmission of MERS disease.

## 5. Conclusions

The present study highlighted the importance of contact network in the spread of infectious disease. These results provide interesting findings. They show that real-time network analysis can provide insight into the structure of transmission of infectivity to identify important players for isolation and selective treatment. Moreover, the results present rational estimate of the size of outbreak and the underlying structural characteristics of the group [37]. Patients with high degree of centrality played a powerful role as epidemic attenuators. Patients with high degree of centrality played a powerful role as epidemic attenuators as they are linked to many secondary cases that did not produce any further secondary cases of their own. While patients with high betweenness are epidemic amplifiers, they are a pathway to infect other patients. This study has shown that most important nodes are those within the hospital, and healthcare workers are more prone to the infection. Pathogenic droplets are easily transmitted within the confined areas of hospitals, and control efforts should be put in place at different layers of hospitals. Reducing contact formation especially within the hospital by restricting hospital visitation for MERS patient families and reducing the number of healthcare workers with access to MERS patients will certainly have significant effect on the spread of MERS disease. Saudi Arabia is said to be facing continuous risk of MERS outbreaks [26]. If control measures are not put in place to curtail the number of hospital visitors and movements of nonessential staff within healthcare facility, with their connectivity to people within the community, the disease will speed up further national/international outbreaks. Future research should focus on epidemiological analysis exploring the role of a healthcare facility in the spread MERS disease.

## Figures and Tables

**Table 1:** Summary of sociodemographic and contact characteristics of MERS-CoV cases in the top 3 infected regions in the KSA during the study period.

Variable	Ar Riyad (*n*=645)	Makkah (*n*=335)	Ash Sharqiyah (*n*=174)	Overall (*n*=1379)	*p* value
Mean age^a^ (SD)	52.73 (19.06)	48.39 (20.01)	61.0 (8.04)	51.7 (18.52)	0.0239
Sex^b^					
Male	373 (57.83%)	226 (67.46%)	125 (71.84%)	901 (65.3%)	<0.0001
Female	263 (40.78%)	100 (29.85%)	49 (28.16%)	460 (33.4%)	
Healthcare worker^c^	87 (13.49%)	60 (17.91%)	23 (13.22%)	203 (14.7%)	<0.0001
Comorbidity^d^	428 (66.4%)	124 (37.0%)	124 (71.13%)	819 (59.4%)	0.2410
Fatal	199 (30.85%)	92 (27.46%)	79 (45.40%)	466 (33.79%)	<0.0001
Place of exposure^e^					
Community linked	48 (7.44%)	6 (1.79%)	17 (9.77%)	81 (5.9%)	<0.0001
Other contacts	66 (10.23%)	94 (28.06%)	27 (15.52%)	224 (16.2%)	
Hospital linked	209 (32.40%)	23 (6.87%)	45 (25.86%)	321 (23.3%)	
Index	0	0	0	2 (0.2%)	
Animal linked^f^	54 (8.37%)	30 (8.96%)	36 (20.69%)	162 (11.74%)	<0.0001
Camel linked^g^	48 (7.44%)	25 (7.46%)	33 (18.97%)	145 (10.51%)	<0.0001

*Note*. ^a^Age: 5 (0.4%) cases missing age value. ^b^Sex: 18 (1.3%) cases of unknown gender. ^c^Healthcare worker: 516 (37.4%) unknown or unclassified healthcare worker status. ^d^Comorbidity: 338 (24.5%), unknown or unclassified comorbidity status. ^e^Contact history: 750 (54.4%) unknown source/place of exposure. ^f^Animal contact: 838 (60.8%) unknown animal contact history. ^g^Camel contact: 867 (62.9%) unknown camel contact history.

**Table 2:** Summary of contact network metrics for the top 10 scores of MERS-CoV KSA during the study period (node (score)).

Score rank	Degree	Betweenness	Closeness	Eigenvector	2-reach
1	1664 (17)	1522 (9)	1664 (0.004)	1664 (1)	1664 (0.068)
2	1025 (6)	1521 (8)	1672 (0.004)	1673 (0.243)	1672 (0.068)
3	124 (5)	897 (7)	1673 (0.004)	1690 (0.243)	1673 (0.068)
4	133 (5)	895 (6)	1674 (0.004)	1693 (0.243)	1674 (0.068)
5	897 (5)	910 (6)	1681 (0.004)	1674 (0.243)	1681 (0.068)
6	898 (4)	1025 (5)	1682 (0.004)	1682 (0.243)	1682 (0.068)
7	1522 (4)	1519 (5)	1683 (0.004)	1687 (0.243)	1683 (0.068)
8	18 (3)	133 (4)	1684 (0.004)	1689 (0.243)	1684 (0.068)
9	98 (3)	996 (2)	1685 (0.004)	1691 (0.243)	1685 (0.068)
10	213 (3)	1008 (2)	1686 (0.004)	1696 (0.243)	1686 (0.068)
